# Gait analysis in a murine model of collagen-induced arthritis

**DOI:** 10.1186/ar2331

**Published:** 2007-11-24

**Authors:** Jon Vincelette, Yifan Xu, Le-Ning Zhang, Caralee J Schaefer, Ronald Vergona, Mark E Sullivan, Thomas G Hampton, Yi-Xin (Jim) Wang

**Affiliations:** 1 Bayer HealthCare Pharmaceuticals, 800 Dwight Way, Berkeley, CA 94701, USA; 2 Mouse Specifics, Inc., 28 State St., Suite 1112, Boston, MA 02109, USA

## Abstract

Murine collagen-induced arthritis (CIA) has become a valuable animal model for elucidating pathogenic mechanisms and evaluating therapeutic effects for rheumatoid arthritis. Recent advances in digital imaging and computer technology have enabled gait analysis to develop into a powerful tool for objectively detecting functional deficits in human and animal models. The present study explored the use of non-invasive video-capture gait analysis in the evaluation of a murine CIA model. CIA was induced in 45 female DBA/1LacJ mice (8 to 10 weeks old) by immunization with lyophilized bovine articular type II collagen. Gait parameters were determined by ventral plane videography and were correlated to traditional arthritis clinical scores. Our results showed that increases in clinical scores that measure the severity of CIA corresponded to changes in multiple gait parameters that reflect both morphologic (increases in paw area) and functional (increase in stride frequency, decrease in stride length, hind-limb paw placement angle, as well as stride, stance, and braking times) deficits. Our work indicated that the non-invasive video-capture device may be used as a simple and objective data acquisition system for quantifying gait disturbances in CIA mice for the investigation of mechanisms and the evaluation of therapeutic agents.

## Introduction

Rheumatoid arthritis (RA) is an autoimmune disease characterized by chronic inflammation in the limbs and joints, cyclic progressive cartilage and bone destruction, and severe disability [1]. The disease has a prevalence of 1% in the adult population worldwide, making RA one of the most common chronic inflammatory diseases [1]. Although different types of treatment can be used to alleviate symptoms, there is no known cure for RA. Further research to understand the pathogenic mechanisms and to develop novel therapeutics, therefore, is necessary [2,3]. Collagen-induced arthritis (CIA) in susceptible strains of mice has become a valuable animal model in RA research because of its simplicity, rapid disease onset, and reproducibility. The availability of transgenic or gene-deficient mice further enhances the power of the CIA mouse model for the investigation of the molecular mechanism of the disease [4]. The most commonly used method for assessing the severity of CIA is a semi-quantitative clinical scoring system based on the degree of inflammatory responses in the paws and joints, which is subjectively determined by the investigator [5-10]. Currently, no established method is available to objectively evaluate the functional abnormality in the mouse CIA model. Gait analysis has been used as a powerful technique in evaluating locomotion in humans and laboratory animals with RA [11]. Recently, ventral plane videographic treadmill gait analysis (DigiGait Imaging System; Mouse Specifics, Inc., Boston, MA, USA) has been proven as a simple, sensitive, and objective method for detecting the gait abnormalities in amyotrophic lateral sclerosis [12] and in Parkinson and Huntington diseases [13] in mice. Therefore, the aim of this study was to evaluate the novel 'objective' gait analysis system in relation to the traditional 'subjective' clinical scoring system in a mouse model of CIA.

## Materials and methods

### Animal model

Forty-five female DBA/1LacJ mice, 8 to 10 weeks old (20 g), were obtained from The Jackson Laboratory (Bar Harbor, ME, USA) and group-housed. All experimental procedures were conducted according to a protocol approved by the Institutional Animal Care and Use Committee. Lyophilized bovine articular type II collagen (purchased from Marie Griffiths, University of Utah, Salt Lake City, UT, USA) was dissolved in 0.01 N acetic acid (2 mg/mL) and kept at 4°C for 24 hours prior to immunization. The type II collagen was mixed with an equal volume of Freund's complete adjuvant (4 mg/mL; Chondrex, Inc., Redmond, WA, USA) on ice, using cold tuberculin syringes and a three-way stopcock. Each mouse was weighed and injected intradermally at the base of the tail with 0.1 mL of emulsion containing 100 μg collagen, using a 1-mL glass tuberculin syringe with a 26-guage needle.

### Clinical scores

The severity of arthritis was scored based on the level of inflammation in each of the four paws and recorded as one of four grades: 0, no symptoms; 1, erythema and edema; 2, joint distortion; and 3, joint ankylosis [5,8]. The average score for each animal was determined by summating the score of all individual limbs and dividing by 4. The clinical score was evaluated weekly over the course of 10 weeks by an experienced investigator who was blinded to experimental groups. To avoid the interruption of the natural progress of the disease by introducing the animals to treadmill walking, a baseline gait analysis was performed only at week 3 following immunization before disease symptoms became evident (clinical score = 0), and an endpoint was taken at the conclusion of the 10-week study when disease severity was high. To analyze the correlation between the clinical score and gait parameters, the individual animals or limbs were further grouped by the severity of the disease measured at the end of the experiment, with average clinical scores of less than or equal to 1 designated as 'mild', less than or equal to 2 as 'moderate', and less than or equal to 3 as 'severe'.

### Gait analysis

**Figure 1:**
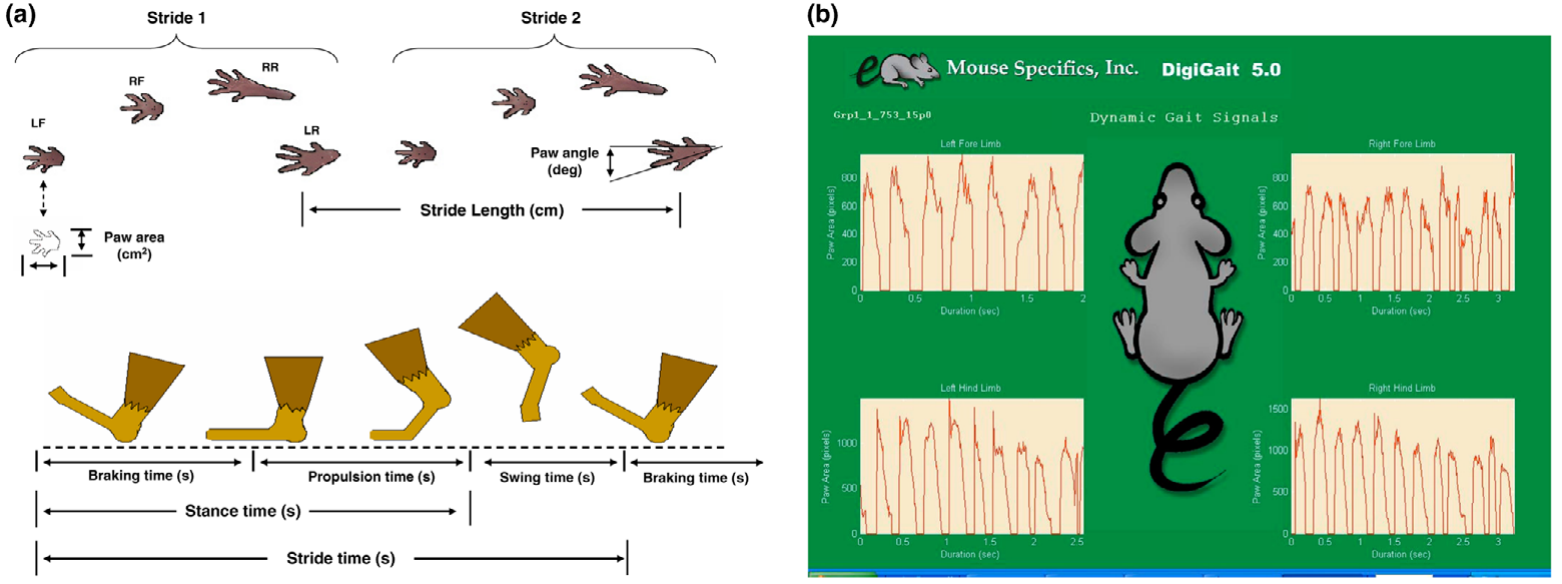
Diagrammatic illustration of gait parameters. **(a) **The ventral view of the test mouse. Computer-digitized paw images used to represent stride length, paw angle, and paw area (left). One complete stride is the summation of the periods in which each paw spends in the phases of stance and swing (right). The stance phase is further divided into braking and propulsion phases. **(b) **Screen-captured image of periodic waveforms representing the advance and retreat of each of four limbs' motion relative to the treadmill belt. LF, left front; LR, left rear; RF, right front; RR, right rear.

Analysis of the ambulatory gait of mice was quantified using the DigiGait Imaging System (Mouse Specifics, Inc.). This system enables mice to walk on a motorized transparent treadmill belt, below which a video camera is mounted to capture the image of the ventral side of the animals. DigiGait automatically pixelates and vectorizes the ventral view of the subject (Figure 1a). The proprietary software and artificial intelligence algorithms analyze the resulting digital images and define the area of each paw corresponding to its movement to generate a set of periodic waveforms that describe the advance and retreat of the four limbs relative to the treadmill belt through consecutive strides (Figure 1b). The software automatically identifies the portions of the paw that are in contact with the treadmill belt as the stance phase of the stride and the portions not in contact with the treadmill belt as the swing phase of the stride (Figure 1a). Numerous postural and kinematic metrics of gait dynamics were determined by dissecting the time each limb is spent in various portions of the walking phase, including paw area, paw placement angle during stance, stride length, stepping frequency, and stance width, as described in detail in Table 1.

**Table 1:** Gait indices and descriptions

Gait index	Description
Paw area	The maximal paw area in contact with the treadmill during the stance phase of the step cycle
Paw angle	The angle of the hind paws in relation to the long axis of the body and its direction of motion
Stride frequency	The average number of times a paw contacts the treadmill belt per second
Stride length	The distance between initial contacts of the same paw in one complete stride
Stride time	The amount of time to complete one complete stride for one limb
Stance time	The portion of the stride in which the paw remains in contact with the belt
Swing time	The forward portion of the stride in which the paw is not in contact with the belt
Braking time	The time between initial paw contact with the belt and the maximal paw contact
Propulsion time	Time between maximal contact and the end of stance, just prior to swing

A range of walking speeds were investigated in a pilot experiment to determine a maximal speed that all animals were able to 'walk', thereby eliminating differences in self-selected speeds as the most important confounder in the interpretation of subjects walking over ground. In this study, the treadmill speed was set at 15 cm/s, at which all of the mice, including those with severe pathology (average clinical score of greater than 2), were able to consistently ambulate for at least four complete strides.

### Statistics

All results are presented as the mean ± standard error of the mean. DigiGait parameters measured at the end of the 10-week study were compared with the baseline value measured at week 3 by means of a paired Student *t *test. Differences were considered statistically significant when the *p *value was less than 0.05.

## Results

### Clinical score

**Figure 2:**
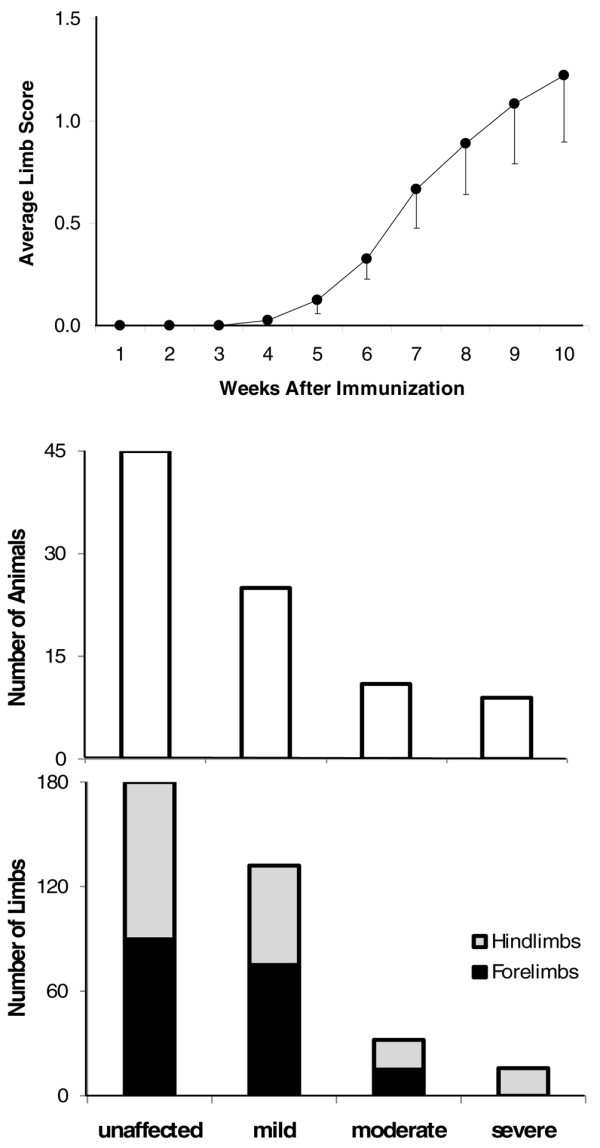
Disease progression over time following collagen immunization as measured by average clinical score of all mice, which was obtained by the summation of the clinical scores of the four limbs in each mouse divided by 4 (top). Distribution of the total number of mice in corresponding disease severity group (middle). Distribution of the total number of limbs in corresponding disease severity group, regardless of the cohort limbs (bottom).

Animals were evaluated weekly throughout the progressive phase of arthritis development. The average clinical scores of animals, which were determined by summating the score of all individual limbs and dividing by 4, started to increase at week 4 and progressively increased over the 10-week time course (Figure 2, top). As described in Materials and methods, the animals were grouped as mild, moderate, and severe based on the clinical score to analyze the correlation between the clinical score and gait parameters, such as stride frequency and stance time and so on. The greatest number of animals were distributed in the mild group (Figure 2, middle). For the parameters of paw area and hind-limb stance angle, which provide information about pathology of the individual limb, each limb was scored individually and pooled by the severity of the clinical score, regardless of cohort limbs. Similarly, the greatest number of limbs were distributed in the mild group (Figure 2, bottom). There was an overall even distribution between fore and hind limbs, except in the severe group, which did not contain fore limbs.

### Gait and posture indices

#### Paw area and angle

**Figure 3:**
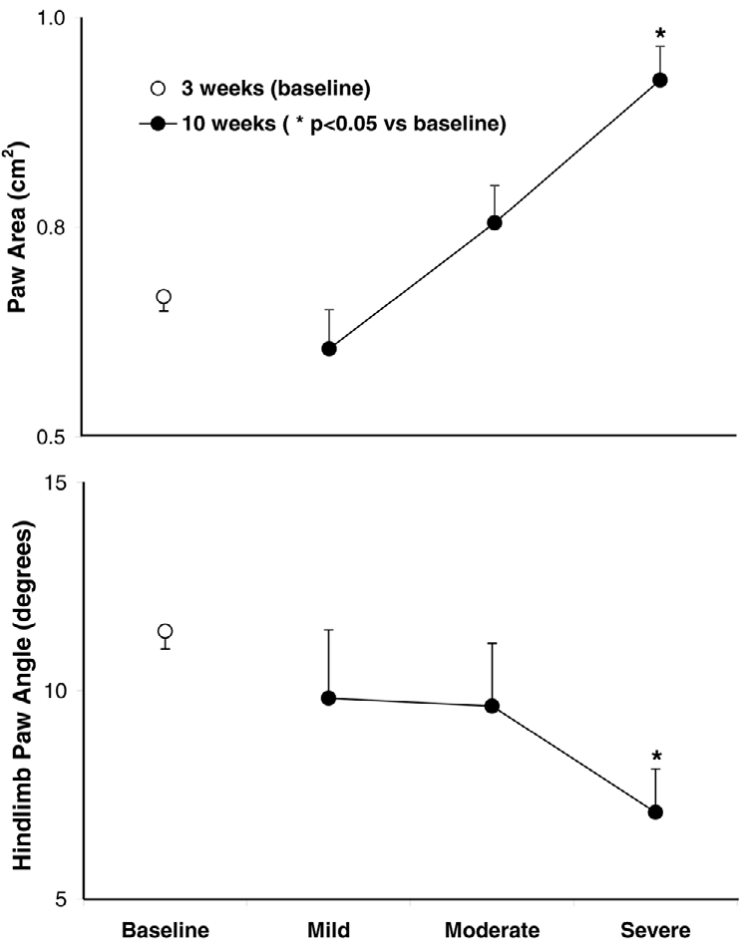
Paw area (top) and hind-limb paw angle (bottom) correspond to disease severity as grouped by clinical score in individual paws. Paw area progressively increased, whereas hind-limb paw angle progressively decreased, with increasing clinical scores in individual paws. Values are means ± standard error of the mean (*p *< 0.05 versus baseline).

The baseline paw area during stance on week 3 before clinical evidence of disease was present was 0.67 ± 0.02 cm^2 ^and exhibited no evident inflammation. This area progressively increased in animals with increasing clinical scores (Figure 3, top). The hind-limb paw angle (the angle of the hind paws during peak stance in relation to the long axis of the body and its direction of motion) showed a baseline value of 11.4 degrees, which also progressively decreased with increasing clinical scores, indicating that those paws with greatest swelling rotated inward during stance to be more parallel with the long axis of the body (Figure 3, bottom).

#### Stride parameters

**Figure 4:**
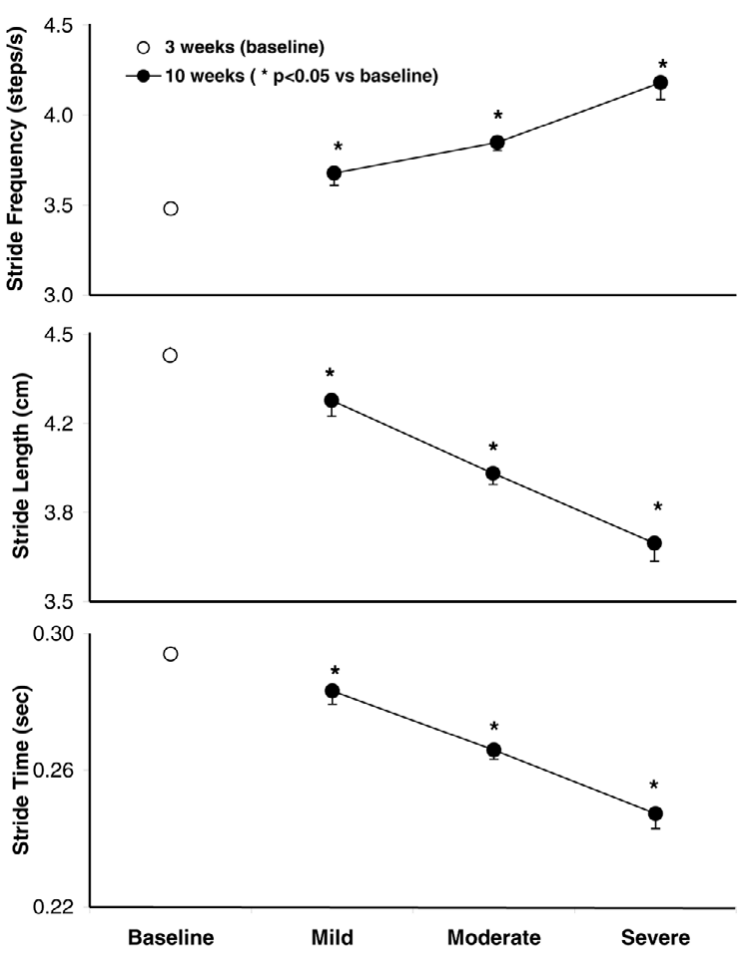
Stride frequency (top), stride length (middle), and stride time (bottom) correspond to disease severity as grouped by average clinical score in individual animals. Stride frequency progressively increased, whereas stride length and time progressively decreased, with increasing clinical scores in individual animals. Values are means ± standard error of the mean (*p *< 0.05 versus baseline).

Stride frequency (the average number of times a paw contacts the belt per second) increased linearly with increasing clinical score (Figure 4, top). Subsequently, with an increase in stride frequency at a fixed walking speed (15 cm/s), stride length (the distance between initial contact of the same paw in a complete stride) (Figure 4, middle) and stride time (Figure 4, bottom) progressively decreased with increasing clinical scores.

#### Phases of the step cycle

**Figure 5:**
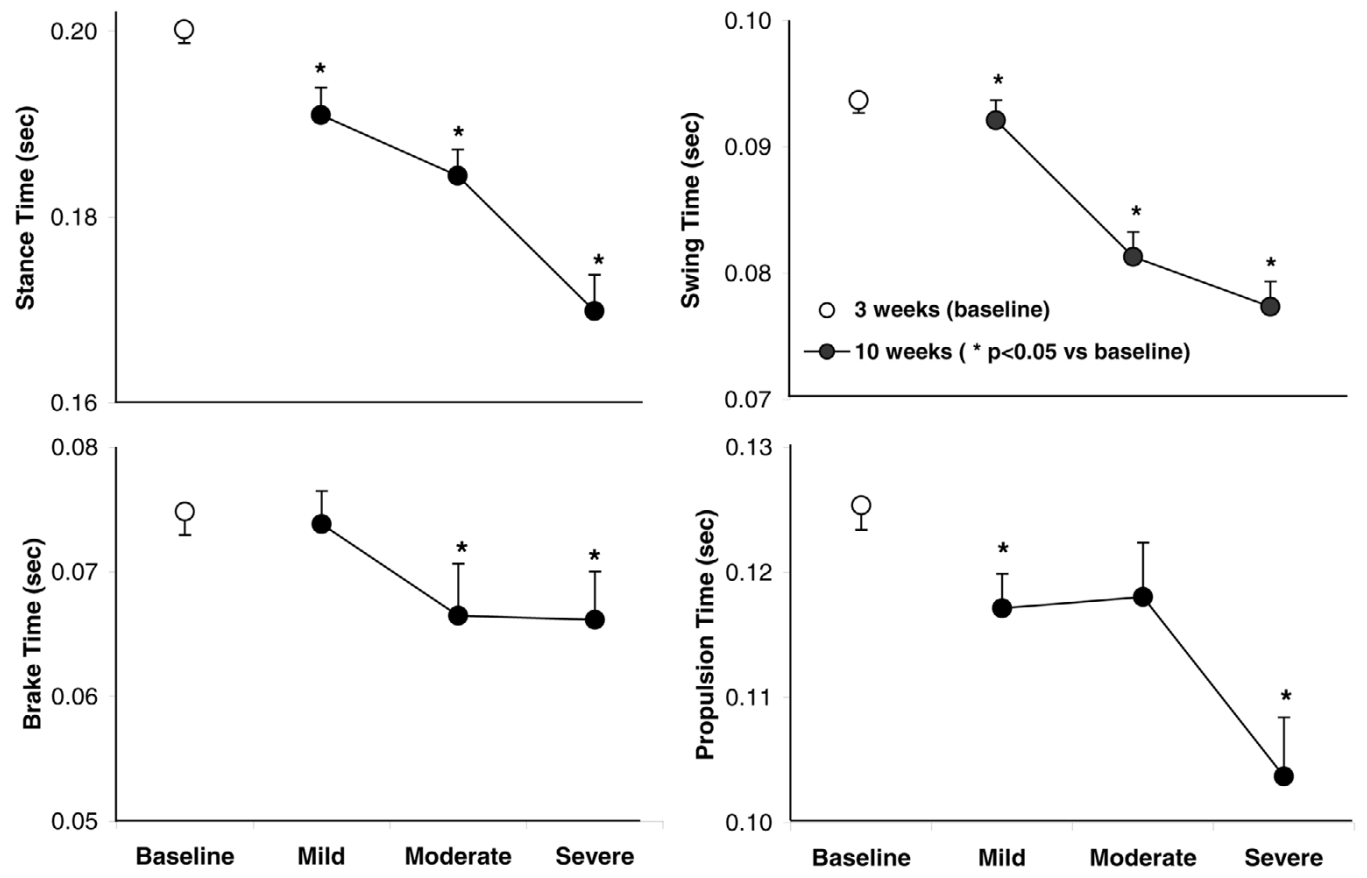
Stance, swing, braking, and propulsion times correspond to disease severity as grouped by average clinical score in individual animals. All four parameters progressively increased with increasing clinical scores in individual animals. Values are means ± standard error of the mean (*p *< 0.05 versus baseline).

One complete stride for a limb is the summation of the stance and swing phases (Figure 5). The stance time (the weight-bearing portion of the stride in which the paw remains in contact with the belt) decreased progressively with increasing clinical scores. A shorter stance time indicates a briefer period in which an inflamed paw or limb is weight-bearing. The swing time (the portion of the stride in which the paw is not in contact with the belt) also decreased progressively with the increase in clinical scores. The stance phase is further divided into the braking and propulsion phases. The braking time (the time interval between initial and maximal paw contacts with the belt) decreased significantly from baseline at the end of the study in animals with greater pathology. The propulsion time (the period between the stance phases and beginning of the swing phase when the paw is not in contact with the belt) decreased progressively with the increasing clinical scores.

## Discussion

The present study explored the use of the non-invasive video-capture gait-analysis instrumentation in the evaluation of a mouse model of CIA. We demonstrated that postural and kinematic gait disturbances corresponded to clinical scores with increasing severity of the CIA. CIA mice exhibited increased paw areas, increased stride frequency, shorter stride length, relative paw placement inversion, and reduced stride, stance, braking, swing, and propulsion durations. To the best of our knowledge, this is the first work seeking to apply comprehensive gait analysis in rodent CIA models.

RA is an autoimmune disease of unknown etiology which leads to chronic inflammation in the joints and subsequent destruction of the cartilage and erosion of the bone [1]. The rodent model of CIA has been proven as a successful animal model for RA research because it is also an autoimmune model and in many ways resembles RA, including the chronic inflammation [4,14]. The primary manifestation of this model is the inflammation-induced joint swelling [15-17]. Although the clinical scoring system may give a numerical score corresponding to the degree of inflammation based on the severity of joint swelling and redness, it provides a semi-quantitative and subjective assessment. Although the degree of inflammation and joint swelling can also be determined by measuring the volume of the paws using a water displacement method in a rat model of CIA [18,19], this method is less practical in mice given the small size and volume of the paws. Using DigiGait, we are able to quantify the maximal paw area in contact with the treadmill during the stance phase of the step cycle and demonstrate that the paw area increased with increasing clinical scores. Thus, the paw area measured digitally by DigiGait is an objective index of tissue swelling and inflammation, which is superior to other parameters of joint inflammation and swelling, such as paw joint thickness [7] and ankle diameter [6], by avoiding experimenter biases.

Ankylosis and joint angle malformation are major pathological changes in CIA, especially at the late stage of the disease [20]. X-ray radiographic analysis has been used to assess this change [6,8]; however, this method is an endpoint outcome, requiring sacrificing the animals, and is also based on a semi-quantitative scoring system [6,8]. In the present study, we used the DigiGait system to measure the paw angle. The measured paw angle corresponds to the severity of the clinical scores. This may reflect either ankylosis or joint angle malformation which forces the placement of paws at these angles or may reflect a conscious compensation for balance or gait disturbances caused by joint pain or inflammation. Thus, to the best of our knowledge, this is the first work indicating that gait analysis by this non-invasive video-capture device could provide a simple alternative way to detect the joint malformation in CIA mice.

To date, the major examination in studies of CIA mouse models includes clinical symptoms as well as radiographic and histological assessments of arthritis. Most of them are endpoint examinations, and there is no method available for assessment of the functional and behavioral defects during the progression of the disease [5-10]. Although an animal's locomotion velocity over ground itself can provide valuable information relating to pain and gait deficits, a change in velocity would make it difficult to analyze the gait parameters. By allowing the investigators to set the treadmill belt speed (15 cm/s), the DigiGait system enables the evaluation and comparison of changes in numerous gait parameters between subjects without the interference of changes in velocity and the requisite changes in gait. In the present study, we measured the stride frequency, stride length, as well as stride, stance, braking, propulsion, and swing times. It has been reported that arthritic changes in the articular surfaces within the joint manifest themselves as an increase in the frequency and a reduction in the length of strides in both human and animal subjects [21]. In the present study, we have observed that the stride frequency indeed increased and the stride length decreased linearly with increasing clinical scores, thus confirming this important observation in the current CIA model. Consistent with these changes, we also observed a linear decrease in stride time. Changes in stride frequency can also be reflected in changes in stance, swing, propulsion, and braking times. Each of these individual parameters comprises one portion of a full stride. Consistent with a decrease in stride time, the time spent in these four phases also decreased linearly with increasing clinical scores. Thus, the present data demonstrate that gait parameters could be used as objective indices for the progression of CIA.

## Conclusion

The present data demonstrate that video-capture gait analysis using the DigiGait system strongly corresponds to measurable pathophysiological changes as determined by the clinical scoring system in a mouse model of CIA. Thus, this non-invasive video-capture device could be used as a simple and objective data acquisition system for quantifying the abnormal movement patterns in CIA mice and evaluating treatment effectiveness.

## Abbreviations

CIA = collagen-induced arthritis; RA = rheumatoid arthritis.

## Competing interests

TGH is employed by Mouse Specifics, Inc., a purveyor of the DigiGait technology applied in this study. All other authors declare that they have no competing interests.

## Authors' contributions

YW and LZ contributed to study design, analysis and interpretation of data, and manuscript preparation. JV contributed to study design, acquisition of data, analysis and interpretation of data, manuscript preparation, and statistical analysis. CJS contributed to study design and manuscript preparation. YX contributed to acquisition of data. TGH contributed to acquisition of data and manuscript preparation. MES and RV contributed to manuscript preparation. JV and YX contributed equally to the work for this manuscript. All authors read and approved the final manuscript.
